# The Prevalence of Musculoskeletal Pain and Assessment of Potential Risk Factors Among a Sample of Medical Students in Giza, Egypt

**DOI:** 10.7759/cureus.70644

**Published:** 2024-10-01

**Authors:** Aly T Aly, Mohamed Hasan, Moaz E Abouelmagd, Shehab M Abouhussain, Mohamed S Mohamed, Mohamed H Mohamed, Abdelrahman W Mansour, Mohamed A Mohamed, Mostafa A Mohamed, Osama Ahmed Abd Elaziz, Hossam Safouh

**Affiliations:** 1 Orthopedics and Traumatology, Misr University for Science and Technology, Giza, EGY; 2 Orthopedics and Traumatology, Medical Research Group of Egypt (MRGE), Cairo, EGY; 3 Neurology, Misr University for Science and Technology, Giza, EGY; 4 Neurology, Medical Research Group of Egypt (MRGE), Cairo, EGY; 5 Neurology, Faculty of Medicine, Cairo University, Cairo, EGY

**Keywords:** back pain, cross-sectional studies, egypt, habits, medical, musculoskeletal pain, neck pain, risk factors, shoulder pain, students

## Abstract

Introduction: Musculoskeletal pain (MSP) is one of the most prevalent conditions among global medical students, affecting their daily lifestyle. In this study, we seek to determine the prevalence of MSP and its associated risk factors in a sample of medical students at Misr University for Science and Technology, Egypt.

Methods: A cross-sectional study was conducted between December 2023 and February 2024, with a sample size of 1472 students who filled out a modified version of the Standardized Nordic Questionnaire. Demographic variables, study hours, and special habits were collected and analyzed. The prevalence of MSP in the past week and year was documented and analyzed using common risk factors.

Results: A total of 971 responses were considered for analysis (66% of the total sample size and 27.7% of the total medical students). The majority were between 18 and 25 years old, with 50.7% males and 49.3% females. The median age was 21 (20-23) years. Most of the students were in their fifth year (23.8%). The prevalence of at least one MSP site was 459 (47.2%) in the past week and 702 (72.2%) in the past year. MSP in the past year was significantly associated with females (OR = 2.45, 95% CI = 1.82-3.3, p = 0.001) and those with a family history of autoimmune disease (OR = 2.19, 95% CI = 1.3-3.5, p = 0.001), while it was significantly associated in the past week with females (OR = 1.72, 95% CI = 1.3-2.2, p = 0.001) and those with a personal history of autoimmune disease (OR = 2.26, 95% CI = 1.09-4.7, p = 0.025). In the past year, neck pain was significantly associated with females (OR = 1.83, 95% CI = 1.42-3.27, p = 0.001) and those with a family history of autoimmune disease (OR = 1.4, 95% CI = 1.01-2.08, p = 0.047). Students living inside October city were less likely to have neck pain in the past year than those who lived outside October city (OR = 0.73, 95% CI = 0.56-0.94, p = 0.017). Shoulder pain in the past year was significantly associated with females (OR = 2.9, 95% CI = 2.1-3.98, p = 0.001) and those with a family history of autoimmune disease (OR = 1.9, 95% CI = 1.28-2.81, p = 0.001) while back pain in the past year was significantly associated with females (OR = 2.1, 95% CI = 1.6-2.7, p = 0.001). Gender was the only factor that was associated with MSP on multivariate regression analysis for the past week (p = 0.001) and past year (p = 0.001).

Conclusion: MSP is highly prevalent among medical students in Egypt, especially females, those with a personal history of autoimmune disease, and those with a family history of autoimmune disease. Despite the high prevalence and the effect on daily lifestyle, very few medical students sought help. More studies are needed to evaluate other risk factors and effective approaches to alleviate MSP among medical students in Egypt.

## Introduction

Medical school can be a challenging experience when creating and training proficient physicians. While physical and mental stressors are a daily routine for medical students, musculoskeletal disorders arise as a consequence of this lifestyle. It poses a significant burden on health issues as there is a high prevalence among the general population, particularly those in the workforce, especially in low and middle-income countries. Hence, the increased economic costs are due to lost productivity and healthcare expenditures. The global burden of musculoskeletal disorders, including pain, is affecting 1.3 billion people worldwide in 2017, with higher prevalence in women and increasing with age [1].

Work-related lower body musculoskeletal pain results in an average of 4.12 hours lost per week per affected worker in the UK. This is an extensive burden on healthcare systems in both developed and developing countries. An analysis in Ontario, Canada reported that musculoskeletal diseases accounted for a significant percentage of outpatient visits and hospitalizations, with a total cost reaching approximately 1.8 billion [2]. The 2014 report on the global disability burden of musculoskeletal diseases ranked it sixth concerning disability-adjusted life years [3,4].

Regarding medical students, musculoskeletal pain turns out to be a relatively common finding, with various studies indicating significant rates across different regions. Research from a public sector university in Karachi, Pakistan found that 74.4% of medical students reported musculoskeletal pain in at least one body site in the past 12 months [5]. In a private Malaysian medical college, the prevalence among medical students was reported as 65.1% at least in one site in the past year, with 45.7% reporting pain in the past week [6].

Academic stress and mental pressure inherent in medical education can further predispose students to musculoskeletal pain [6-10]. The association between musculoskeletal pain and psychological stress is well documented, indicating that students experiencing musculoskeletal pain also suffer from increased levels of anxiety and stress. This psychological distress can further deteriorate sleep quality, creating a vicious circle where poor sleep contributes to a heightened perception of pain, which in turn exacerbates psychological distress [11].

Chronic pain, including musculoskeletal pain, is associated with decreased concentration and physical discomfort during studying and clinical activities [12]. Subsequently, the burden on medical students’ academic functioning and clinical performance can be severe [13].

Thus, addressing the physical and psychological components of musculoskeletal pain in medical students is crucial for their overall health and academic success. Concerning that overall documentation and evidence are lacking in Egypt, and the effect of musculoskeletal pain is overlooked, this study aims to investigate the prevalence of musculoskeletal pain among medical students at Misr University for Science and Technology, a private university in Egypt, and analyze the associated possible risk factors.

## Materials and methods

This study was reported according to the Strengthening the Reporting of Observational Studies in Epidemiology (STROBE) statement guidelines [14]. The Institutional Review Board at Misr University for Science and Technology (MUST-IRB) approved the study on 27 December 2023 (approval number: 2022/110). MUST-IRB is registered at the Office for Human Research, US Department of Health and Human Services, and operates under federal-wide assurance number FWA00025577. It was conducted according to the ethical principles of the Declaration of Helsinki. Participation in this study was completely voluntary. An informed consent question was present at the start of the questionnaire, which had to be accepted to take the survey. The informed consent explained the purpose of the study and assured privacy and anonymity. It also explained that the answers given in the questionnaire would only be used for academic and clinical research purposes and that personal data were securely stored and only accessible to the statistician. Refusal of the informed consent question would result in the termination of the questionnaire, with no further questions asked.

Study design, setting, and participants

We conducted a cross-sectional study between December 2023 and February 2024. We targeted medical students of both sexes at our medical college. We included medical students as well as internship students. We excluded graduates, dentistry students, physical therapy students, pharmaceutical students, nursing students, students of applied medical sciences, non-medical students, and medical students from other private or governmental medical colleges. We implemented convenience sampling by using an online, self-administered questionnaire hosted on Google Forms (Google, Mountain View, CA). The questionnaire link was shared in all medical classes' official educational communication groups.

Variables

The study variables were sociodemographic information, including age, gender, marital status, height in centimeters, weight in kilograms, academic year, and residence, whether inside the 6th of October city or outside, as well as factors that might be associated with musculoskeletal pain such as coffee consumption, special habits, history of arthritis or other autoimmune diseases, family history of autoimmune diseases, study hours, screen hours, working hours per week, type of physical activity, and training hours.

Data sources and measures

The online survey was adopted from the "Standardized Nordic Questionnaire" as the one used by Smith et al. [15,16], with added adjustments and modifications, which enabled more tailored findings for our study. A professional translator assisted us in translating the questionnaire to culturally appropriate Arabic, retranslated it back to English, and compared it to the initial one to check for consistency.

The questionnaire was composed of three major sections: the first section included sociodemographic questions, location, and the total number of musculoskeletal pain sites (neck, right and left shoulders, right and left elbows, right and left wrists and hands, lower back, hips, knees, ankles, and feet). The second section included different risk factors associated with musculoskeletal pain such as a history of arthritis or other autoimmune diseases, family history of autoimmune disease, daily study hours and screen time hours, working hours per week, special habits, and physical sports. The third section included focused questions for neck, shoulder, and lower back pain such as persona, history of pain in a lifetime, personal history of pain in the past week and past year, hospital admissions, and lifestyle changes due to pain.

Pilot and validity

A pilot study was conducted, which contained a sample of 50 medical students to ensure the validity, reliability, and complete understanding of the questionnaire. It showed a Cronbach’s α of 0.788, which was acceptable. We obtained participant feedback by conducting a direct interview after completing the questions.

Bias

Based on the pilot study, the estimated average time to complete the questionnaire was three to five minutes. Responses that were completed in less than three minutes and those completed in more than five minutes were excluded from the analysis. We used timestamps for both the start and submission times to determine the completion time for each participant. Additionally, an external analyst reviewed the analysis to help minimize potential bias. Data from the pilot study were not included in the final analysis reported in this manuscript.

Sample size and power calculation

The primary outcome of this study is assessing the prevalence of musculoskeletal pain in medical students and associated risk factors. Previous data from the literature suggest that 54% of students (at least in one site) reported musculoskeletal pain in the past week [17]. To detect a similar effect with a 5% absolute deviation and 95% confidence level, a minimum sample of 345 participants was required for this study, considering that the medical college has about 3500 enrolled students.

Statistical methods

Data from the questionnaire were extracted into an Excel spreadsheet (Microsoft Corporation, Redmond, WA) and encoded to be compatible with the statistics software. Data analysis was performed using Jamovi software v 2.3.28. A descriptive analysis was done to acquire the frequencies, mean, and standard deviation. For each body site studied, the frequency and percentage of musculoskeletal pain during the previous week and year were noted. This study contained two dependent variables: “musculoskeletal pain in the past week at least in one site” and “musculoskeletal pain in the past year at least in one site.” The χ2 test was used to calculate the crude odds ratio (OR) and 95% confidence interval (CI) to evaluate the association between the categorical independent factors and the dependent variables. Every single one of the dependent variables was subjected to a separate multivariate binary logistic regression analysis. P < 0.05 was considered statistically significant.

## Results

Participants

We obtained complete responses from 1472 participants and excluded 501 responses that did not meet the minimum average completion time (three to five minutes); hence, a total of 971 responses were included in the final analysis.

Sociodemographic characteristics

We found that 478 (49.3%) of the respondents were females. A total of 530 (54.5%) respondents were in their clinical years, while 440 (45.5%) were in their pre-clinical years. The median age was 21 (20-23) years, and the median BMI was 24.7 (22.3-27.5). The majority of participants (528, 54.4%) regularly played sports. Of the participants, 50 (5.2%) were cigarette smokers, while 51 (5.3%) smoked both cigarettes and sheesha. A total of 135 (13.9%) of the participants had a family history of autoimmune disease, while 33 (3.4%) had a personal history of autoimmune disease. The sociodemographic characteristics of all the participants who completed the questionnaire are presented in Table 1.

**Table 1:** Demographic characteristics of the participants.

Variables	Median (IQR) or N (%)
Age in years	21 (20-23)
Height in cm	170 (162-177)
Weight in kg	70 (61-81)
BMI	24.7 (22.3-27.5)
Gender	
Male	492 (50.7%)
Female	479 (49.3%)
Residence	
Inside October city	576 (59.3%)
Outside October city	395 (40.7%)
Study year	
1	156 (16.1%)
2	132 (13.5%)
3	153 (15.8%)
4	128 (13.2%)
5	231 (23.8%)
6	91 (9.4%)
Internship	80 (8.2%)
Clinical status	
Pre-clinical	440 (45.5%)
Clinical	531 (54.5%)
Coffee consumption	
>3 cups per day	101 (10.4%)
<3 cups per day	512 (52.7%)
Never	358 (36.9%)
Special habits	
Cigarette smokers	50 (5.2%)
Sheesha smokers	27 (2.8%)
Cigarette and sheesha smokers	51 (5.3%)
Alcoholics	3 (0.3%)
All the above	6 (0.6%)
None of the above	834 (85.9%)
Personal history of autoimmune disease	
Yes	33 (3.4%)
No	938 (96.6%)
Family history of autoimmune disease	
Yes	135 (13.9%)
No	836 (86.1%)
Study hours per day	
<2 hours	130 (13.4%)
2-4 hours	321 (33.1%)
4-6 hours	310 (31.9%)
>6 hours	210 (21.6%)
Screen hours per day (mobile, tablet, or computer)	
<2 hours	17 (1.8%)
2-4 hours	121 (12.5%)
4-6 hours	233 (23.9%)
>6 hours	600 (61.9%)
Working hours per week	
<35 hours	51 (5.3%)
35-56 hours	50 (5.2%)
>56 hours	16 (1.6%)
Not working	854 (87.9%)
Marital status	
Single	955 (98.4%)
Married	16 (1.6%)
Sports	
Weightlifting	137 (14.1%)
Football	121 (12.5%)
Tennis	27 (2.8%)
Swimming	23 (2.4%)
Basketball	18 (1.9%)
Jogging	49 (5.0%)
Other sports	153 (15.8%)
None	443 (45.6%)
Hours spent training per week	
1-5	322 (33.2%)
5-10	142 (14.6%)
10-15	45 (4.6%)
>15	18 (1.9%)
None	444 (45.8%)
Playing music	
Yes	60 (6.2%)
No	911 (93.8%)

Prevalence of musculoskeletal pain

The prevalence of musculoskeletal pain in at least one body site was 462 (47.2%) in the past week and 710 (72.3%) in the past year. A total of 412 (42.5%) students who had a history of musculoskeletal pain reported having problems with daily life activities (Table 2).

**Table 2:** Prevalence of musculoskeletal pain (MSP) sites.

Variables	Past week	Past 12 months
MSP (general)	462 (47.6%)	710 (73.1%)
Neck	213 (22%)	413 (42.5%)
Back	206 (21%)	353 (36.3%)
Shoulder	125 (13%)	224 (23.1%)
Elbow	34 (3.5%)	70 (7.2%)
Wrist	115 (11.9%)	170 (17.5%)
Hip	44 (4.5%)	90 (9.3%)
Knees	78 (8%)	178 (18.3%)
Ankles	112 (11.5%)	123 (12.7%)
None	509 (52.4%)	261 (26.9%)
Number of pain sites		
0-1	786 (81%)	550 (56.7%)
2-5	170 (17.5%)	371 (38.2%)
5-11	15 (1.5%)	50 (5.1%)

Among the specific pain sites, neck pain was the most common, with a prevalence of 213 (22%) in the past week and 413 (42.5%) in the past year, followed by lower back (206 (21%) and 353 (36.3%)) and shoulders (125 (13%) and 224 (23%)). Lower back pain reported the highest hospital admissions (45, 4.6%), while neck pain was the most correlated with changes in lifestyle activities (22, 22.7%) (Table 3).

**Table 3:** Neck, shoulder, and lower back pain and their specific associations.

Variables	Neck	Lower back	Shoulder
History of pain in a lifetime	481 (49.6%)	412 (42.5%)	296 (30.5%)
Hospital admission due to pain	23 (2.3%)	45 (4.6%)	29 (2.9%)
Job changes due to pain	43 (4.4%)	77 (8%)	39 (4%)
Pain causing physical therapy/physician visits in the past 12 months	60 (6.1%)	74 (7.6%)	45 (5%)
Pain duration in the past 12 months			
0 days	557 (57.4%)	617 (64%)	746 (77%)
1-7 days	244 (25.2%)	201 (20.5%)	132 (13.6%)
8-30 days	49 (5%)	52 (5.3%)	29 (3%)
>30 days but not daily	61 (6.2%)	59 (6%)	35 (3.6%)
Daily	59 (6%)	41 (4.2%)	28 (2.8%)
Pain causing lifestyle changes in the past 12 months	221 (22.7%)	212 (21.9%)	135 (14%)
Duration of lifestyle changes in the past 12 months			
0 days	788 (81%)	785 (81%)	855 (88%)
1-7 days	135 (14.2%)	125 (13%)	75 (7.5%)
8-30 days	30 (3%)	25 (2.5%)	20 (2.25%)
>30 days	17 (1.7%)	35 (3.5%)	20 (2.25%)

The prevalence of musculoskeletal pain in the past week was higher among females (OR = 1.7, 95% CI = 1.3-2.2, p < 0.001) and among those with a personal history of autoimmune disease (OR = 2.26, 95% CI = 1.09-4.7, p = 0.025). Musculoskeletal pain prevalence in the past year was also higher among females (OR = 2.45, 95% CI = 1.82-3.50, p < 0.001) and among those who had a family history of autoimmune disease (OR = 2.19, 95% CI = 1.34-3.56, p = 0.001) (Table 4).

**Table 4:** Factors associated with musculoskeletal pain (MSP) during the past week and past year among medical students using the chi-squared test.

		MSP during the past week				MSP during the past year			
Variables		Yes	No	OR (95% CI)	p-value	Yes	No	OR (95% CI)	p-value
										Gender	Male	201 (21%)	291 (30%)	1.72 (1.3-2.2)	<0.001	319 (33%)	174 (17.9%)	2.45 (1.82-3.30)	<0.001
Female	261 (27%)	218 (22.4%)			391 (40.3%)	87 (9%)				
Academic year	Pre-clinical	224 (23%)	217 (22.3%)	1.27 (0.98-1.63)	0.067	320 (33%)	121 (12.5%)	0.95 (0.71-1.27)	0.72
	Clinical	238 (24.5%)	292 (30%)			390 (40.2%)	140 (14.4%)		
Residence	Inside October city	274 (28.2%)	302 (31.1%)	1 (0.77-1.29)	0.99	419(43.1%)	157 (16.2%)	1.05 (0.78-1.4)	0.78
	Outside October city	188 (19.3%)	207 (21.3%)			291 (30%)	104 (10.7%)		
Personal history of autoimmune disease	Yes	22 (2.3%)	11 (1.2%)	2.26 (1.09-4.7)	0.025	29 (3%)	4 (0.04%)	2.74 (0.9-7.8)	0.052
	No	440 (25%)	498 (51.3%)			681 (70%)	257 (26.5%)		
Family history of autoimmune disease	Yes	71 (7.3%)	64 (6.5%)	1.26 (0.87-1.82)	0.2	114 (11.7%)	21 (2.2%)	2.19 (1.34-3.56)	0.001
	No	391 (40.3%)	445 (46%)			596 (61.4%)	240 (24.7%)		
Study hours per day	<2 hours	60 (6.1%)	71 (7.3%)		0.03	98 (10%)	33 (3.4%)		0.57
	2-4 hours	148 (15.2%)	173 (17.9%)			236 (24.3%)	85 (8.7%)		
	4-6 hours	136 (14%)	174 (18%)			231 (23.8%)	79 (8.1%)		
	>6 hours	118 (12.1%)	91 (9.4%)			145 (15%)	64 (6.6%)		
Marital status	Single	456 (47%)	500 (51.5%)	1.37 (0.48-3.87)	0.554	702 (72.3%)	254 (26.2%)	2.42 (0.86-6.74)	0.081
	Married	6 (0.6%)	9 (0.9%)			8 (0.8%)	7 (0.07%)		
Coffee intake	>3 cups per day	59 (6%)	42 (4.3%)		0.06	74 (7.6%)	27 (2.8%)		0.89
	<3 cups per day	235 (24.2%)	278 (28.6%)			378 (3.9%)	135 (13.9%)		
	Never	168 (17.3%)	189 (19.4%)			258 (26.5%)	99 (10.2%)		
Special habits	Cigarette smokers	16 (1.6%)	34 (3.5%)		0.004	36 (3.7%)	14 (1.44%)		<0.001
	Sheesha smokers	14 (1.4%)	13 (1.3%)			21 (2.2%)	6 (0.6%)		
	Cigarette and sheesha smokers	13 (1.3%)	38 (3.9%)			26 (2.7%)	25 (2.6%)		
	Alcoholics	1 (0.1%)	1 (0.1%)			0 (0%)	2 (0.2%)		
	All the above	4 (0.4%)	2 (0.2%)			3 (0.3%)	3 (0.3%)		
	None	414 (42.6%)	421 (43.4%)			624 (64.3%)	211 (21.7%)		
Work hours per week	<35 hours	25 (2.6%)	27 (2.8%)		0.789	43 (4.4%)	9 (0.09%)		0.03
	35-56 hours	24 (2.5%)	26 (2.7%)			39 (4%)	11 (1.1%)		
	>56 hours	6 (0.6%)	11 (1.1%)			8 (0.8%)	9 (0.9%)		
	Not working	407 (42%)	445 (45.8%)			620 (64%)	232 (24%)		
Screen hours per day	<2 hours	5 (0.5%)	12 (1.2%)		0.265	9 (0.9%)	8 (0.8%)		0.307
	2-4 hours	54 (5.5%)	67 (6.9%)			89 (9.1%)	32 (3.3%)		
	4-6 hours	106 (11%)	127 (13%)			172 (17.7%)	61 (6.3%)		
	>6 hours	297 (30.6%)	303 (31.2%)			440 (45.3%)	160 (16.5%)		
Sports	Yes	246 (25.3%)	282 (29%)	1.09 (0.84-1.4)	0.501	389 (40.1%)	139 (14.3%)	0.94 (0.7-1.25)	0.67
	No	216 (22.2%)	227 (23.4%)			321 (33.1%)	122 (12.6%)		
Playing music	Yes	29 (3%)	31 (3.2%)		0.904	44 (4.5%)	16 (1.6%)	1.01 (0.56-1.83)	0.969
	No	433 (44.6%)	478 (49.2%)	0.96 (0.5-1.63)		666 (68.6%)	245 (25.2%)		
BMI	<18.5	19 (2%)	18 (1.9%)		0.187	27 (2.8%)	10 (1%)		0.193
	18.5-24.9	222 (23%)	250 (25.8%)			335 (34.5%)	137 (14.1%)		
	25-29.9	179 (18.4%)	175 (18%)			273 (28.1%)	81 (8.3%)		
	>30	42 (4.3%)	66 (6.8%)			75 (7.7%)	33 (3.4%)		

The prevalence of neck pain in the past year was higher in females (OR = 1.83, 95% CI = 1.42-2.37, p < 0.001), among those who had a family history of autoimmune disease (OR = 1.4, 95% CI = 1.01-2.08), p = 0.047), and among those with a previous history of autoimmune disease (OR = 2.13, 95% CI = 1.05-4.3, p = 0.033). It is worth noting that those who live inside October city were less likely to experience neck pain in the past year than those who lived outside October city (OR = 0.73, 95% CI = 0.56-0.95, p = 0.017). Shoulder pain prevalence in the past year was higher in females (OR = 2.9, 95% CI = 2.11-3.98, p < 0.001) and among those with a positive family history of autoimmune disease (OR = 1.9, 95% CI = 1.28-2.81, p = 0.001) and previous history of autoimmune disease (OR = 2.89, 95% CI = 1.4-5.8, p = 0.002). Lower back pain prevalence in the past year was higher among females (OR = 2.1, 95% CI = 1.6-2.7, p < 0.001) (Table 5).

**Table 5:** Factors associated with neck, shoulder, and lower back pain during the past year among medical students using the chi-squared test.

		Neck pain				Shoulder pain				Lower back pain			
Variables		Yes	No	OR (95% CI)	p-value	Yes	No	OR (95% CI)	p-value	Yes	No	OR (95% CI)	p-value
Gender	Male	173 (17.8%)	319 (32.9%)	1.83 (1.42-2.37)	<0.001	70 (7.2%)	423 (43.6%)	2.9 (2.11-3.98)	<0.001	138 (14.2%)	354 (36.6%)	2.1 (1.6-2.7)	<0.001
	Female	240 (24.6%)	239 (24.6%)			155 (16%)	323 (33.3%)			215 (22.1%)	264 (27.1%)		
Academic year	Pre-clinical	194 (20.1%)	246 (25.3%)	0.881 (0.6-1.14)	0.333	104 (10.7%)	337 (34.7%)	0.959 (0.7-1.29)	0.782	167 (17.2%)	273 (28.2%)	0.88 (0.68-1.15)	0.37
	Clinical	219 (22.47%)	312 (32.1%)			121 (12.5%)	409 (42.1%)			186 (19.1%)	345 (35.4%)		
Residence	Inside October city	227 (23.4%)	349 (36%)	0.73 (0.56-0.95)	0.017	137 (14.1%)	439 (45.2%)	0.9 (0.6-1.25)	0.585	209 (21.5%)	367 (37.8%)	1.01 (0.77-1.31)	0.957
	Outside October city	186 (19.1%)	209 (21.5%)			88 (9%)	307 (31.6%)			144 (14.8%)	251 (25.9%)		
History of autoimmune disease	Yes	20 (2.1%)	13 (1.3%)	2.13 (1.05 - 4.3)	0.033	15 (1.5%)	18 (1.8%)	2.89 (1.4 - 5.8)	0.002	17 (1.7%)	16 (1.6%)	1.9 (0.94- 3.8)	0.065
	No	393 (40.5%)	545 (56.2%)			210 (21.6%)	728 (75%)			336 (34.6%)	602 (62%)		
Family history of autoimmune disease	Yes	68 (7.01%)	67 (6.9%)	1.4 (1.01 -2.08)	0.047	46 (4.7%)	89 (9.2%)	1.9 (1.28-2.81)	0.001	59 (6%)	76 (7.8%)	1.43 (0.9-2.07)	0.056
	No	345 (35.5%)	491 (50.6%)			179 (18.4%)	657 (67.7%)			294 (30.3%)	542 (55.9%)		
Study hours per day	<2 hours	62 (6.4%)	69 (7.1%)		0.069	34 (3.5%)	97 (1%)		0.876	49 (5%)	82 (8.4%)		0.553
	2-4 hours	145 (14.9%)	176 (18.1%)			74 (7.6%)	247 (25.4%)			107 (11%)	214 (22.1%)		
	4-6 hours	133 (13.7%)	177 (18.2%)			70 (7.2%)	240 (24.7%)			120 (12.4%)	190 (19.6%)		
	>6 hours	73 (7.5%)	136 (14%)			47 (4.8%)	162 (16.7%)			77 (7.9%)	132 (13.6%)		
Marital status	Single	409 (42.1%)	547 (56.4%)	0.48 (0.15-1.54)	0.2	224 (21.1%)	732 (75.4%)	0.233 (0.03-1.78)	0.127	349 (40%)	607 (62.5%)	0.63 (0.2-2)	0.4332
	Married	4 (0.4%)	11 (1.1%)			1 (0.1%)	14 (1.3%)			4 (0.4%)	11 (1.1%)		
Coffee intake	>3 cups per day	40 (4.1%)	61 (6.3%)		0.81	20 (2%)	81 (8.3%)		0.66	32 (3.3%)	69 (7.1%)		0.46
	<3 cups per day	221 (22.8%)	292 (30.1%)			119 (12.2%)	394 (40.6%)			194 (20%)	319 (32.9%)		
	Never	152 (15.6%)	205 (21.1%)			86 (8.8%)	271 (27.9%)			127 (13.1%)	230 (23.7%)		
Special habits	Cigarette smokers	19 (2%)	31 (3.2%)		0.489	7 (%)	43 (4.4%)		0.428	19 (1.9%)	31 (3.2%)		0.131
	Sheesha smokers	12 (1.2%)	15 (1.5%)			8 (0.8%)	19 (1.9%)			8 (0.8%)	19 (1.9%)		
	Cigarette and sheesha smokers	27 (2.8%)	24 (2.5%)			9 (0.9%)	42 (44.3%)			10 (1%)	41 (0.42%)		
	Alcoholics	0 (0%)	2 (0.2%)			0 (0%)	2 (0.2%)			0 (0%)	2 (0.2%)		
	All the above	2 (0.2%)	4 (0.4%)			1 (0.1%)	5 (0.5%)			2 (0.2%)	4 (0.4%)		
	None of the above	353 (36.4%)	482 (49.5%)			200 (20.5%)	635 (65.4%)			314 (32.3%)	521 (52.7%)		
Work hours per week	<35 hours	20 (2.1%)	31 (3.3%)		0.7	9 (0.9%)	43 (4.4%)		0.323	21 (2.2%)	30 (3.2%)		0.12
	35-56 hours	18 (1.9%)	32 (3.3%)			16 (1.6%)	34 (3.5%)			15 (1.5%)	35 (3.6%)		
	>56 hours	7 (0.7%)	9 (1%)			3 (0.3%)	14 (1.4%)			2 (0.2%)	14 (1.5%)		
	Not working	369 (37.9%)	485 (49.9%)			197 (20.3%)	655 (67.5%)			315 (32.4%)	538 (55.3%)		
Screen hours per day	<2 hours	4 (0.4%)	13 (1.3%)		0.015	3 (0.3%)	14 (1.4%)		0.423	1 (0.1%)	16 (1.6%)		0.054
	2-4 hours	44 (4.5%)	77 (7.9%)			33 (3.4%)	88 (9%)			44 (4.5%)	77 (7.9%)		
	4-6 hours	87 (9%)	146 (15%)			59 (6%)	174 (17.9%)			81 (8.3%)	152 (15.6%)		
	>6 hours	278 (28.6%)	332 (34.2%)			130 (13.4%)	470 (48.4%)			227 (23.4%)	373 (38.4%)		
Sports	Yes	210 (21.6%)	318 (32.7%)	0.78 (0.6-1.01)	0.057	113 (11.6%)	415 (42.8%)	0.8 (0.5-1.08)	0.153	180 (18.5%)	348 (35.9%)	0.8 (0.6-1.05)	0.109
	No	203 (20.9%)	240 (24.7%)			112 (11.5%)	331 (34.1%)			173 (17.8%)	270 (27.8%)		
Playing music	Yes	22 (2.3%)	38 (3.9%)	0.7 (0.4-1.32)	0.343	12 (1.2%)	48 (5%)	0.8 (0.4-1.5)	0.548	28 (2.9%)	32 (3.3%)	1.58 (0.9-2.67)	0.08
	No	391 (40.3%)	520 (53.6%)			213 (21.9%)	698 (71.9%)			325 (33.5%)	586 (60.4%)		
BMI	<18.5	15 (1.6%)	22 (2.3%)		0.334	11 (1.1%)	26 (2.7%)		0.2	11 (1.1%)	26 (2.4%)		0.823
	18.5-24.9	192 (19.8%)	280 (28.8%)			110 (11.3%)	362 (37.3%)			172 (17.7%)	300 (30.1%)		
	25-29.9	164 (16.9%)	190 (19.6%)			87 (8.9%)	267 (27.5%)			132 (13.6%)	222 (22.9%)		
	>30	42 (4.3%)	66 (6.8%)			17 (1.7%)	91 (9.4%)			38 (3.9%)	70 (7.2%)		

Analysis of the relationship

On multiple logistic regression analysis, factors associated with musculoskeletal pain during the past year were gender (OR = 2.6, 95% CI = 1.8-3.7, p = 0.001), family history of autoimmune disease (OR = 1.83, 95% CI = 1.1-3.06, p = 0.02), those with BMI between 25 and 29.9 (OR = 1.4, 95% CI = 1.02-2.06, p = 0.03), and students who worked part-time jobs (OR = 1.9, 95% CI = 1.02-3.83, p = 0.04). The total model was not significant (Table 6).

**Table 6:** Multiple logistic regression of factors associated with musculoskeletal pain during the past year.

Variable		B	p-value	OR	95% CI
Gender	Females – males	0.184	0.001	2.6	1.8-3.7
BMI	<18.5 – 18.5-24.9	0.2773	0.328	1.312	0.7-2.26
	25-29.9 – 18.5-24.9	0.1768	0.034	1.455	1.02-2.06
	>=30 – 18.5-24.9	0.2543	0.523	1.176	0.7-1.94
Family history of autoimmune disease	Yes – no	0.2606	0.02	1.837	1.1-3.06
Job status	Working – not working	0.3371	0.043	1.977	1.02-3.83

Factors associated with musculoskeletal pain in the past week were gender (OR = 1.7, 95% CI = 1.2-2.4, p <0.001) and coffee consumption of more than three cups per day (OR = 1.92, 95% CI = 1.17-3.1, p = 0.009) (Table 7). The total model was not significant.

**Table 7:** Multiple logistic regression of factors associated with musculoskeletal pain during the past week.

Variable		B	p-value	OR	95% CI
Gender	Female – Male	0.16	<0.001	1.733	1.2-2.4
Drinking coffee	>3 cups/day – no intake	0.2486	0.009	1.92	1.17-3.1
	<3 cups/day – no intake	0.1469	0.786	0.961	0.72-1.2

## Discussion

Key results

This study showed that 47.2% of musculoskeletal pain occurred at least one site in the past week and 72.3% in the past 12 months. Surprisingly, 17.5% and 38.2% of participants reported experiencing pain in two to five sites in the past week and the past 12 months, respectively. It is worth mentioning that the prevalence rates of neck and lower back pain were nearly identical in the past week, at 22% and 21%, respectively. Despite the high impact of neck and lower back pain on lifestyle changes (22.7% and 21.9%), medical students showed no interest in seeking physician advice or undergoing physical therapy.

Moreover, gender was shown to be a significant factor to be associated with pain in general, as females (49.3%) had a higher prevalence of musculoskeletal pain than males. Students who lived inside October city where the university was located had a lower prevalence of neck pain in the past year than those who lived outside October city (OR = 0.73, 95% CI = 0.56-0.94, p = 0.017).

The results indicated that demographics and lifestyle factors such as gender and family history of autoimmune disease are strongly associated with musculoskeletal outcomes. The chi-square test revealed a significant association, particularly between gender, residence, and family history of autoimmune disease and neck and shoulder pain.

Significance of the study

This study expands the literature by providing new data on the experienced musculoskeletal pain among medical students in Egypt at Misr University For Science and Technology. This study showed lower rates among medical students in seeking physician’s advice or physiotherapy care. Therefore, more health education tools are necessary to encourage further investigation of this issue among Egyptian medical students, as evidence is lacking, and to provide better understanding and better care for future physicians.

Agreement and disagreement with previous studies

Concerning the prevalence of musculoskeletal pain, our results are highly consistent with a recent study on medical students by Torbey et al., which found that half of the students experienced pain in the past seven days, compared to 82% in the previous year [17]. The association between gender and musculoskeletal pain in our study is consistent with Torbey et al. and Hendi et al. on medical students in Saudi Arabia and a study in Brazil that reported 77.6% of female medical students had lower back pain [17,18]. AlShagga et al., in Malaysia, showed that the gender factor was insignificant [6]. In our investigation, the areas with the highest frequency of musculoskeletal pain in the previous week and previous year were the neck, lower back, and shoulders. When compared to recent and relatively recent studies by Torbey et al. [17] in Syria and Dighriri et al. [11] in Saudi Arabia, neck pain was reported to be ranked second, with lower back pain being second [19]. However, this was inconsistent with Algarni et al. [7]. Additionally, we observed that neck, shoulder, and lower back pain had a negative effect on lifestyle in general at around 22.7%, 21.9%, and 14%, respectively, which is less than in the study by Torbey et al. [17].

We did not find a significant association between musculoskeletal pain and participants' academic years in the past seven days and 12 months, respectively. Our findings correspond with Torbey et al. [17]. However, a prospective study among medical students in France identified third-year medical students as an independent prognostic factor for lower back pain [20]. Similarly, in Alshagga et al.'s study, musculoskeletal pain in the past year was significantly associated with clinical years and the past week in academic years [6]. In a study among Chinese medical and dental students [21], the prevalence of neck and lower back pain was significantly different among academic years, with a higher prevalence noted in later years.

Our study showed different observations regarding the occurrence of musculoskeletal pain and family history of autoimmune diseases. The situation was significantly different among students who reported pain in the past year. The difference was only significant in the neck and shoulder when analyzed in individual pain sites. This does not correspond with other studies that suggest that inheritance factors may play a role [17,22].

Our findings regarding screen hours were aligned with previous research, including studies by Torbey et al. [17] and Saudi studies, which showed that it had no bearing on the likelihood of developing musculoskeletal pain [9,16,19,23]. However, when analyzed on individual pain sites, there was a significant association with neck pain in the past 12 months. A study by Salameh et al. [9] showed that 75.9% of medical students experienced at least one type of musculoskeletal pain, predominantly in the shoulder and neck, during distance learning during the COVID-19 pandemic.

Interpretation (impact on public health)

Our findings may be the results of a demanding academic environment with prolonged study and clinical setting hours, which contribute significantly to musculoskeletal pain. Students often spend extended periods in static posture, such as sitting or standing, which subsequently can lead to musculoskeletal strain. Moreover, psychological stress and ergonomic setups play a crucial role.

It appears that the lower prevalence of musculoskeletal pain among medical students in low and middle-income countries compared to higher-income countries can be attributed to multiple factors.

Cultural differences may influence how pain is reported, as cultural perceptions may interfere with reporting pain or seeking medical advice [24]. In addition, medical students in these regions might have more responsibilities outside of their academic work, which in turn can lead them to overlook pain.

Moreover, it is important to consider psychological stressors specific to low-income countries, as medical students there face different academic and lifestyle pressures compared to their counterparts, which can impact musculoskeletal pain.

Finally, the higher prevalence of musculoskeletal pain in females is multi-factorial, involving increased psychological stress, prolonged stable postures, biological differences, higher domestic workloads, and poor posture habits. Investigation of these factors could help decrease the burden of musculoskeletal pain in this population [25].

Generalizability

Due to convenience sampling methods, this study's results might have limited generalizability. We attempted to compensate for non-random sampling by increasing the sample, which might draw a random sample from the population. Because medical students are more involved in health issues, reporting of lifestyle changes and pain rates might be exaggerated. We modified the questionnaire to meet our culture; hence, the results cannot be generalized to a worldwide population.

Strengths and limitations

To our knowledge, this study is one of the earliest works investigating the prevalence of musculoskeletal pain in medical students, thus understanding how far musculoskeletal pain can impact academic and clinical performance and the lifestyle of medical students. Our study has multiple points of strength, as we collected a relatively large sample size and used a standardized questionnaire that was spread among medical students only.

This study used a self-administered online-based questionnaire on medical students in only one private sector university. We also excluded students from other medical specialties, such as veterinary medicine, dentistry, physical therapy, and nursing. We did not use a standardized questionnaire to measure physical activity. Although we tried to limit biases by including responses that were done between three and five minutes only, there is a concern for sampling bias due to convenience sampling and self-report bias.

## Conclusions

Our study suggests that musculoskeletal pain is a highly prevalent condition among medical students in Giza, Egypt. The neck, shoulders, and lower back were the most commonly associated sites. Significant factors associated with musculoskeletal pain were female gender, personal history of autoimmune disease, and family history of autoimmune disease. According to the study results, health awareness campaigns regarding musculoskeletal pain among students are needed due to lower rates of affected individuals seeking care. Universities should consider promoting physical activities, providing resources for managing stress, and performing regular pain checkups on medical students. Further studies are required to assess the prevalence among other medical schools in Egypt and to understand better the causality and long-term effects of these risk factors on musculoskeletal pain.
